# Modelling strategic interventions in a population with a total fertility rate of 8.3: a cross-sectional study of Idjwi Island, DRC

**DOI:** 10.1186/1471-2458-12-959

**Published:** 2012-11-08

**Authors:** Dana R Thomson, Michael B Hadley, P Gregg Greenough, Marcia C Castro

**Affiliations:** 1 Harvard Medical School, Boston, MA, 02115, USA; 2 Department of Global Health and Population, Harvard School of Public Health, Boston, MA, 02115, USA; 3 Harvard Humanitarian Initiative, Harvard University, Cambridge, MA, 02138, USA

## Abstract

**Background:**

Idjwi, an island of approximately 220,000 people, is located in eastern DRC and functions semi-autonomously under the governance of two kings (*mwamis*). At more than 8 live births per woman, Idjwi has one of the highest total fertility rates (TFRs) in the world. Rapid population growth has led to widespread environmental degradation and food insecurity. Meanwhile family planning services are largely unavailable.

**Methods:**

At the invitation of local leaders, we conducted a representative survey of 2,078 households in accordance with MEASURE DHS protocols, and performed ethnographic interviews and focus groups with key informants and vulnerable subpopulations. Modelling proximate determinates of fertility, we evaluated how the introduction of contraceptives and/or extended periods of breastfeeding could reduce the TFR.

**Results:**

Over half of all women reported an unmet need for spacing or limiting births, and nearly 70% named a specific modern method of contraception they would prefer to use; pills (25.4%) and injectables (26.5%) were most desired. We predicted that an increased length of breastfeeding (from 10 to 21 months) or an increase in contraceptive prevalence (from 1% to 30%), or a combination of both could reduce TFR on Idjwi to 6, the average desired number of children. Increasing contraceptive prevalence to 15% could reduce unmet need for contraception by 8%.

**Conclusions:**

To meet women’s need and desire for fertility control, we recommend adding family planning services at health centers with NGO support, pursuing a community health worker program, promoting extended breastfeeding, and implementing programs to end sexual- and gender-based violence toward women.

## Background

**Figure 1:**
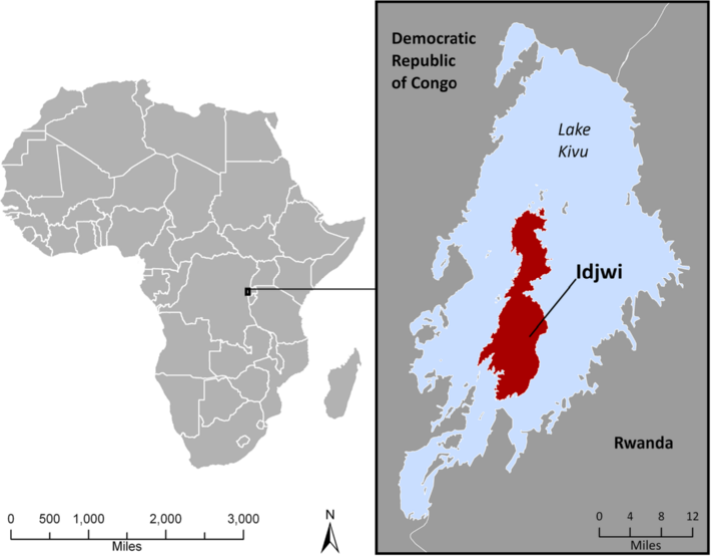
Location of Idjwi, Democratic Republic of the Congo, Africa.

Idjwi, Africa’s 2^nd^ largest inland island (106 sq. mi.), is located in Lake Kivu in eastern Democratic Republic of Congo (DRC) on the periphery of DRC’s civil conflict (Figure 1). While family planning services have become widely available across Africa, they remain nearly absent on Idjwi. With a total fertility rate (TFR) of 8.3 births per woman, and roughly 2,075 people living per square mile, high population density and rapid population growth are often associated with environmental degradation and major nutritional deficiencies on Idjwi.

In 2010, at the invitation of Idjwi’s leaders, a multi-disciplinary team from Harvard’s public health, medical, policy, and design schools performed a health needs assessment to guide improvements to Idjwi’s health infrastructure, and attract attention and resources to the island’s increasingly dire health situation. To provide community leaders, government officials, and aid organizations with actionable information, this health assessment included a population-based household survey, key informant interviews, focus groups with vulnerable subpopulations, and the creation of the first cartographic map of the island. To the best of our knowledge, the household survey was the first of its kind on Idjwi, both in terms of the breadth of health topics covered and its representativeness of communities across the island.

This comprehensive assessment identified several urgent health problems; among the most urgent was lack of family planning services. Not only are sexual and reproductive health services core to the provision of public health [1], a lack of family planning services is intimately related to several other identified health concerns including sexual- and gender-based violence against women [2], poor child health [3], and household food insecurity from population pressure [4].

### Health, population and fertility

Few sources of information about Idjwi health conditions were available before this assessment. Published studies identified extremely high fertility rates [5] and endemic goiter prevalence [6] in the 1970s, low HIV seroprevalence in the 1980s [7], and low healthcare utilization in the 1990s [8]. Missionary groups described worse health outcomes among the island’s minority Batwa (Pygmy) population compared to the general population [9], and international advocacy groups reported high maternal mortality, poor infant and child health, malnutrition, high rates of infectious diseases, and poor health infrastructure [10,11]. A national Demographic and Health Survey (DHS) conducted in the DRC in 2007 did not include Idjwi [12].

**Figure 2:**
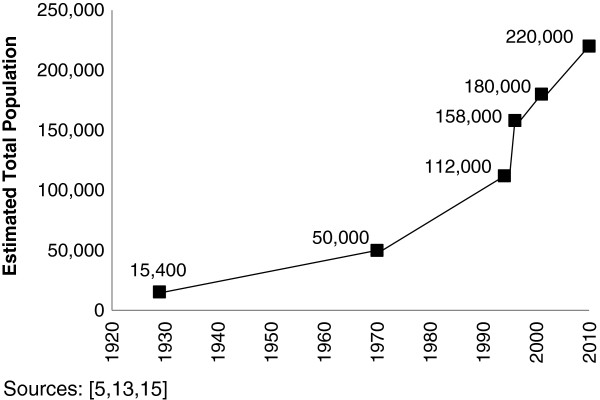
Historical population trend, Idjwi, DRC.

Limited demographic data show alarming population trends for Idjwi over the last century. Consistently high fertility and immigration, combined with the island’s fertile soil and regular rainfall (55 ± 8 inches annually) [5] supported annual population growth rates ranging from 2.9% to 3.3% between 1929 and 1994 (Figure 2) [5,13]. In the 1950s, TFR in South Kivu province was 8.5, and it has remained high since [14]. During the 1994 Rwandan Genocide, Idjwi’s population increased 41% when 46,000 Rwandan refugees, mostly women and children, fled to the island [13]. Since the mid-1990’s, many Congolese have also moved to Idjwi fleeing the conflict in mainland DRC. In 2001, LandScan population estimates put Idjwi’s population just under 180,000 [15]. Based on these trends and an annual growth rate of 2.2% since 1994, we estimated Idjwi’s population to be approximately 220,000 in 2010. Satellite imagery from 2009 [16] and field observations in 2010–2012 revealed extensive deforestation.

### Barriers to health and family planning

Many factors limit the provision of family planning services on Idjwi. The island’s health personnel, infrastructure, supplies, financing, and outreach programs are insufficient to meet local needs. The health system receives no support from the Congolese government and relies entirely on user fees and foreign assistance. In interviews, local health practitioners acknowledge the need for family planning, but said they were reluctant to discuss the topic with patients if their practices had no regular supply of contraceptives. Additionally, most women visit health centers only for perinatal or emergent care, and many rely entirely on traditional and home remedies. As a result, the health system provides few women with family planning options or information (Table 1).

**Table 1:** Women’s reproductive health and empowerment experiences, Idjwi, DRC, 2010

**Variable**	**N**	**Weighted mean or percent**	**(95% CI)**	**Missing**
Desired number of children (mean)	2078	6.1	(6.0, 6.2)	13
Age of first intercourse (mean)	2078	16.8	(16.7, 16.9)	31
Age started living with husband/partner (mean)	2078	17.7	(17.6, 17.8)	157
Months exclusively breastfed last child (mean)	2027	6.1	(5.9, 6.3)	661
Births attended by skilled health personnel (%)	2027	66.0	(63.6, 68.3)	22
Attended at least one antenatal care visit (%)	2027	84.8	(83.0, 86.6)	90
Ever used any modern contraceptive (%)	2078	6.5	(5.4, 7.6)	36
Of modern contraceptive users: Male condom	140	12.5	(6.7, 18.3)	--
Of modern contraceptive users: Pill	140	50.6	(41.5, 59.7)	--
Of modern contraceptive users: Injections	140	40.3	(31.4, 49.2)	--
Of modern contraceptive users: Other	140	10.0	(4.4, 15.6)	--
Ever used withdrawal method (%)	2078	2.5	(1.8, 3.2)	36
Ever used calendar method (%)	2078	3.7	(2.9, 4.5)	36
Unmet need for contraception (%)	2078	53.8	(53.4, 54.2)	7
Unmet need for limiting births	2078	15.5	(13.8, 17.2)	--
Unmet need for spacing births	2078	38.3	(36.1, 40.5)	--
Women in polygynous marriage (%)	2078	14.6	(13.8, 17.2)	154
Self-reported literacy (%)	2078	37.8	(35.5, 40.1)	20
Health worker spoke to respondent about family planning in the last year (%)	2078	14.2	(12.9, 16.3)	9
Respondent’s health care decisions are made by ___ (%)				147
Herself	2078	6.9	(5.7, 8.1)	--
Husband/partner	2078	85.2	(83.5, 86.9)	--
Herself and husband/partner	2078	7.0	(5.8, 8.2)	--
Someone else	2078	0.9	(0.4, 1.3)	--
Respondent believes it is “common” or “very common” for a woman in her community to be…				
Beaten by her husband/partner	2078	52.3	(50.0, 54.7)	11
Verbally threatened by her husband/partner	2078	48.1	(45.8, 50.5)	12
Forced by her husband/partner to have sex	2078	39.4	(37.1, 41.7)	17
Forced by someone other than her husband/partner to have sex	2078	35.6	(33.4, 37.9)	19
Respondent believes a husband is justified to beat wife if…				
She goes out without telling him	2078	80.4	(78.5, 82.3)	31
She neglects the children	2078	76.0	(74.0, 78.0)	33
She argues with him	2078	68.3	(66.1, 70.5)	36
She refuses to have sex with him	2078	69.9	(67.7, 72.1)	36
She burns the food	2078	57.8	(55.5, 60.1)	35

Sociopolitical factors also limit access to family planning. In focus groups, women argued that poverty, limited education, geographic isolation, patrilineal inheritance, normalization of violence, lack of formal justice systems, and absence of women in government all buttress social conservatism and suppress women’s reproductive rights. In other similar settings, women’s disempowerment in terms of limited decision making power within intimate relationships [17,18], violence against women [19], and low education [20] limited access to reproductive health services, and promoted high fertility. Furthermore, half of the island’s population is devout Catholic, and local priests maintain that condoms, injections, and other “unnatural” forms of contraception are forbidden.

### Fertility transition

Evidence of high fertility and high mortality on Idjwi indicates that this population has not yet entered a fertility transition. Fertility transitions are generally proceeded by a reduction in child mortality [21,22], and are accompanied by improvements to health and economic conditions including women’s increased access to education and economic opportunities [23]. Access to contraception, especially among young women, is critical for woman to be able to control fertility and leverage educational and economic opportunities [24]. In the case of Idjwi, access to contraception is not only a geographic and economic challenge, but also a social one; effective family planning programs put women solely in charge of their own fertility decisions which may have previously been influenced by husbands and extended family [25]. In societies where women have limited control over their own fertility, practicing extended periods of breastfeeding can help to lower fertility levels [26]. Where violence against women is widespread, programs that educate men about reproductive health, and engage men in conversations about gender equity have made headway in promoting women’s reproductive health and empowerment [27].

In this context of poverty, high fertility, and social marginalization, we investigated women’s needs and desires for family planning, and modeled scenarios to meet these needs based on proximate determinants of fertility. We discuss results and make evidence-based, actionable recommendations drawing on the literature as well as key informant interviews and focus-groups conducted in Idjwi communities.

## Methods

### Household survey data

**Figure 3:**
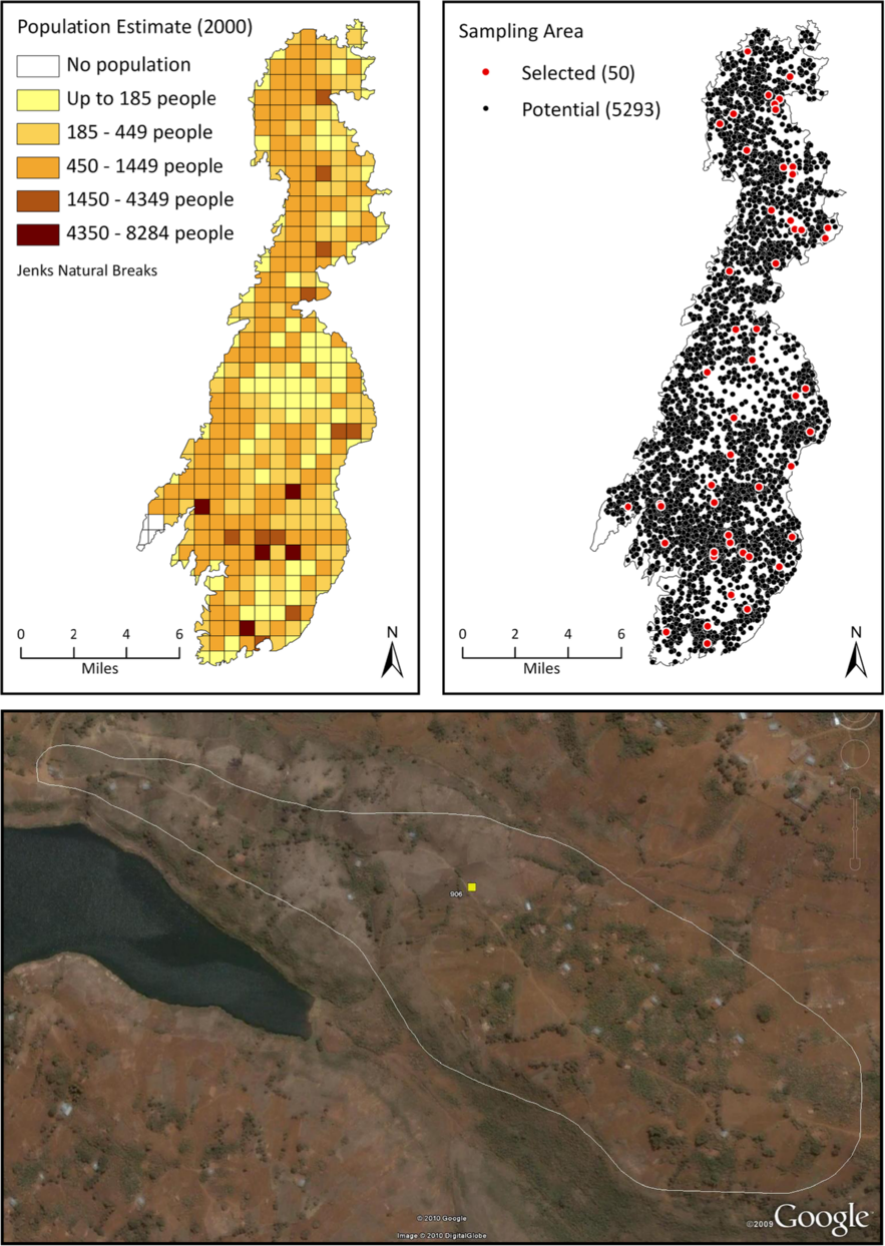
**Cluster sampling approach.** (Top left) LandScan population grid. (Top right) Coordinates placed in proportion to population density, and 50 coordinates randomly selected. (Bottom) Example of sampling area delineated around the ~ 45 closest dwellings to a selected coordinate in satellite imagery.

We conducted a representative household survey between June and August 2010 of 2,078 women age 18 to 50 from 50 randomly selected sampling units across Idjwi. We used LandScan population estimates [15] and satellite imagery [16] to select a one-stage cluster sample. LandScan estimates were generated by an algorithm that allocates census figures to one square kilometer grid cells based on land surface characteristics identified by satellite images. Using ArcGIS 9.3 (ESRI, Redlands, CA), we generated 5,293 geographic coordinates in proportion to the population density in each of the 383 grid cells, resulting in 1 to 242 coordinates per grid cell. We then randomly selected 50 coordinates without replacement, allowing two or more coordinates to fall in the same grid cell. Using Google Earth, we delineated sampling areas around the approximately 45 closest dwellings to each selected coordinate, and used this information to create a field navigation map of roads, paths, and sampling units (Figure 3). In the field, interviewers started at the center of each sampling area and performed interviews at every household encountered within the sampling area boundary, regardless of accessibility. We aimed to complete 45 interviews in each sampling area, although the actual number ranged from 40 to 48.

Eligible households were defined as having one female resident between the age of 18 and 50. A number of households were headed by a woman under the age of 18 and could not be interviewed per the internal review board (IRB) requirements of our study. If the household had more than one eligible woman, the interviewer used a simplified KISH table [28] to select one woman at random. If the selected woman was not present or unable to complete the interview, the interviewer followed up with the woman at an agreed upon time. The timing of the survey during Idjwi’s dry season meant that women were more likely to be at home rather than working in the fields. No respondents refused to participate.

The household survey was modeled on the Standard MEASURE DHS Women’s and Household Questionnaires [29,30] with additional items incorporated from the WHO World Health Survey Household and Individual Questionnaires [31], the Women’s Health Study of Accra Questionnaire [unpublished], and the Harvard School of Dental Medicine Global Oral Health Survey [unpublished]. The resulting Idjwi demographic and health survey was approved by the Harvard School of Public Health Office of Human Research Administration and by Idjwi officials.

Following MEASURE DHS guidelines [32], we trained 41 local female interviewers age 17 to 27, and seven local male and female survey team leaders. The survey was administered verbally in Ki’Havu, the only language that is spoken by all people on Idjwi. Questions were translated from English to Ki’Havu and then back translated by two independent groups of English teachers. The Ki’Havu version was tested on Idjwi, and modified for clarity to reflect the range of local responses. For potentially distressing topics such as domestic violence and rape, we asked respondents about general problems in their community rather than their personal experiences. We collected complete birth histories from each respondent, along with prenatal and postnatal care information, household characteristics, individual socioeconomic characteristics, and reproductive health practices and beliefs. Throughout the survey process, we met with leaders (the island’s two *mwamis,* the archbishop of South Kivu, and village chiefs) and citizens to explain the purpose of the survey and address questions and concerns.

We employed sampling weights in our analysis to adjust for differences in the number of households interviewed in each sampling unit. Since some households had multiple eligible women, additional weights were applied in the calculation of women’s health indicators. Weights were calculated as:

(1) $$Household\;weight=\frac{1}{\left(\frac{c*50}{C}\right)\left(\frac{i}{\text{P/h}}\right)}$$

(2) $$Women\;weight=Household\;weight*\frac{1}{w}$$

where:

*c* = number of selected coordinates in LandScan cell

*C* = total potential coordinates

*i* = number of households interviewed in LandScan cell

*P* = total estimated population in LandScan cell

*h* = average household size in LandScan cell

*w* = number of women in household

### Key informant data

In June through August of 2010, 2011 and 2012, we performed interviews and focus groups with key informants and members of vulnerable subpopulations to understand the socio-political context, potential drivers of poor health outcomes, and perspectives about how to improve health. Key informant interviews were iterative and relied on snowball sampling to identify local leaders in government, agriculture, trade, education, health care, religion, civics, women’s rights, justice, and security. Focus groups also used snowball sampling to identify 20 groups of 8–12 individuals from vulnerable groups across the island, including women, Batwa (pygmies), and communities far from health services. Interviews and focus groups were conducted in Ki’Havu by a member of the research team assisted by a translator from Idjwi. Sessions focused on determinants and solutions to problems identified by participants. All participants were over age 18, and gave free and informed consent.

### Primary outcomes: preference and need for family planning

**Figure 4:**
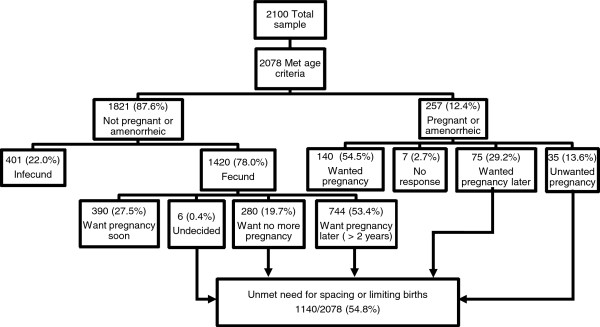
Schematic of fertility desires and unmet need for contraception, Idjwi, DRC, 2010.

There are two main outcomes of interest. The first, “preference for contraception,” is the percent of women who said they would like to use a specific method of contraception if given the option [33]. The second, “unmet need for contraception,” is a widely accepted measure of demand for contraception that would exist in a society if all women had geographic, economic, and social access to family planning services [25]. A woman is considered to have an unmet need for family planning if she wishes to space or limit her births now or in the near future but is unable [33] (see Figure 4).

### Secondary outcomes: proximate determinants of fertility

Demographers model fertility using proximate determinants through which more distal factors, such as women’s empowerment and education, affect the timing and number of births [34]. We use Stover’s five fertility indices [35] based on Bongaarts’ seminal work [36] to understand the relative influence of each proximate determinant on Idwi’s current fertility level, and to assess how hypothetical scenarios of increased in contraceptive use and breastfeeding could reduce future fertility rates. The indices are defined and measured as follows; the closer the index value to zero, the more influence that proximate determinant has on fertility in the population.

A. *Sexual activity index* (*Cx)* is the percent of women who report being sexually active or have had vaginal intercourse in the last month.

B. *Infecundity index* (*Cf)* is derived from the number of sexually active women who are fecund, which includes women who have had a pregnancy in the last five years, are not pregnant, and are not experiencing post-partum lactational amenorrhea (*f*).

C. *Postpartum infecundability index* (*Ci)*, is based on *i*, the number of months a woman reported lactational amenorrhea after her last pregnancy. In our survey, we did not ask about lactational amenorrhea, and instead of total months of breastfeeding, we asked about exclusive breastfeeding. As lactational amenorrhea is closely linked with the period of exclusive breastfeeding, we used exclusive breastfeeding as a proxy for the length of postpartum infecundability.

D. *Current contraceptive prevalence index* (*Cu)* is the percent of women currently using modern or traditional contraception, and the average effectiveness of those methods. In the Idjwi survey, we asked women if they had ever used modern or traditional methods of contraception. Because contraception is essentially unavailable, we did not ask about current use. Less than 7% of women reported ever using any form of modern contraception (Table 1), and even fewer reported ever using withdrawal (2.5%) or the calendar method (3.7%). For modeling of proximate determinants, we used a generous estimate of 1% current contraception prevalence (*u*), and an average effectiveness rate (*e*) of 95% suggested by Stover [28].

E. *Induced abortion* (*Ca)* is the fifth proximate determinate in Stover’s model, though it is difficult to measure in household surveys because women tend to under-report induced abortion due to strong social stigma and laws prohibiting the practice [37]. Abortion is illegal in DRC except to save the life of the woman, and is not offered at any health facilities on Idjwi. Many demographers believe that induced abortion is rare to non-existent in high-fertility, resource-poor settings [38]. Previous research [5] and our qualitative work found that abortion, although rare and strongly disregarded, is practiced on Idjwi, often with the assistance of traditional healers. Interviews with leadership of UFIN, Idjwi's premier women's rights organization, suggest that "at least 1%" of women have had unsafe abortions, and that most women know how to get an abortion. Assuming that women who have induced abortions have only one, the induced abortion index is 0.9988, which rounds to 1, and conforms to standard modeling assumptions that the abortion rate is essential zero.

Stover’s proximate determinants model also includes a *potential fertility* (*PF*) term equal to 21 which is the theoretical maximum number of children a fecund woman could physically bear if she remained sexually active the entire time between ages 15 and 49 with no constraints on her fertility. The model of total fertility is:

(3) TFR=Cx*Cf*Ci*Ca*Cu*PF

Of the five proximate determinates, contraceptive prevalence and length of breastfeeding can be feasibly altered with family planning programs and policies. Using this model, we assess the effect of moderate increases in contraceptive use and longer periods of breastfeeding on TFR and women’s unmet need for family planning.

## Results

Table 2 summarizes household characteristics for this sample of 2,078 women. Although households earn a monthly average US$60.97 purchasing power parity (PPP) in hard currency, the disparity between the poorest and wealthiest households is striking. The wealthiest 20% of households earn US$293.25 PPP in hard currency each month, 1,600 times more than the poorest 20% of households which earn just US$0.18 PPP in hard currency each month. These figures do not include land holdings and other forms of non-monetary wealth, which suggests that the disparity between the richest and poorest is likely greater. Chronic food insecurity exists in 26% of households, and periodic food shortages are a problem in another third.

**Table 2:** Household characteristics, Idjwi, DRC, 2010

**Variable**	**Weighted mean or percent**	**(95% CI)**	**Missing**
Monthly Household Income in USD (mean)*	$60.97	($43.51, $78.43)	888
Lowest Quintile	$0.18	($0.14, $0.22)	--
2^nd^ Lowest Quintile	$2.07	($1.93, $2.21)	--
Middle Quintile	$7.30	($7.03, $7.57)	--
2^nd^ Highest Quintile	$17.30	($16.32, $18.28)	--
Highest Quintile	$293.25	($206.91, $379.59)	--
Household Nutrition in last year (%)			33
Enough to eat, and kinds of food we want	14.6	(13.0, 16.1)	--
Enough to eat, NOT kinds of food we want	26.4	(24.4, 28.3)	--
Sometimes don’t have enough/anything to eat	32.1	(30.0, 34.2)	--
Often don’t have enough/anything to eat	25.9	(23.9, 27.9)	--

N=2078.

* Households that trade more goods than hard currency might appear poorer in this table than they actually are.

### Reproductive health and empowerment

Given existing fertility trends on Idjwi, a woman who survives adulthood is expected to have 8 births throughout her lifetime (TFR=8.3). Table 1 summarizes women’s reproductive health outcomes and empowerment experiences. Contraceptive use is extremely low. Of women who have ever used a modern method (6.5%), most have used pills (50.6%) or injectables (40.3%). Only 14% of women reported that a health worker talked to them about family planning in the last year. Women reported exclusively breastfeeding for an average of 6.1 months, compared to just 1.7 months in the rest of South Kivu province [12]. Using Bongaarts’ equation relating length of breastfeeding to post-partum infecundability [36], we estimated that Idjwi women breastfeed for a total of 10.4 months. Women’s reproductive lives start early. Sixteen percent of women reported having their first intercourse between ages 10 and 14, and the average age at first intercourse was 16.8 years. On average, women moved in with their first husband/partner and had their first child before age 18, which constitutes statutory rape under Congolese law. Thirty percent of women reported having sex before moving in with their husband/partner. Fifteen percent of women were in polygynous marriages.

We also asked women about their understanding of violence in the community. Forty percent reported it was common or very common for a woman to be forced by her husband/partner to have sex, and 36% reported it was common or very common for a woman to be forced into sex by someone other than her husband/partner. In interviews, leaders of Idjwi’s premier women’s organization, UFIN, estimated that 70% of married women have been physically beaten by their husbands. They added that “many women are beaten to the point where they needed medical attention,” sometimes so badly that they “risk miscarrying their fetus”. Violence against women appears to be a community norm. The majority of survey respondents believed a husband is justified to beat his wife if she goes out without telling him (80.4%), neglects the children (76.0%), argues with him (68.3%), refuses to have sex with him (69.6%), or burns the food (57.8%). These figures are comparable, and in some cases worse than, mainland DRC [12].

### Preference for contraception

We asked women about the total number of children they wished to have and desired use of contraceptives, though these were likely perceived as abstract questions given how little control women have over their own fertility. Women reported wanting 6.1 children on average, approximately two children less than the current fertility level. Seven in ten women named a specific modern method of contraception they would prefer to use if given the option; most said pills (25.4%) or injections (26.5%) (Table 3).

**Table 3:** Preferred method of contraception

**Type**	**Weighted percent**	**(95% CI)**
Injection	26.5	(24.4, 28.5)
Pill	25.4	(23.3, 27.4)
Don’t want to use	20.4	(18.5, 22.4)
Other	9.9	(8.5, 11.5)
Spermicide (foam/jelly)	6.1	(5.0, 7.2)
Don’t know	4.9	(3.8, 6.0)
IUD	3.4	(2.5, 4.2)
Withdrawal	3.3	(2.4, 4.3)

N=2078, missing=57.

### Unmet need for contraception

Figure 4 outlines the criteria that resulted in 1,140 fecund, pregnant, or amenorrheic women with an unmet need for contraception. Women who reported an unmet need for spacing or limiting future births were asked to explain why they were not currently using a method of contraception. Women were able to give up to 20 pre-coded reasons, as well as describe other reasons (later coded), or simply say “I don’t know.” Over 900 women gave at least one reason for not using contraception and most women gave two to five reasons (Table 4). The most common reasons cited were currently breastfeeding (21.7%), cost too much (20.5%), not knowing where or how to obtain contraception (18.9%), not knowing which contraceptive methods are available (18.3%), husband’s opposition to use of contraception (15.9%), and fear of side effects from taking contraception (15.6%).

**Table 4:** Reasons women with unmet need do not use contraception

**Reason**	**Weighted percent**	**(95% CI)**
Breastfeeding/postpartum amenorrhea	21.7	(18.8, 24.6)
Costs too much	20.5	(17.6, 23.4)
Knows no source	18.9	(16.1, 21.6)
Knows no method	18.3	(15.5, 21.0)
Husband opposed	15.9	(13.3, 18.5)
Health concern/fear side effect	15.6	(13.0, 18.1)
Fatalistic	14.0	(11.6, 16.4)
Interferes with body’s processes	7.2	(5.5, 8.9)
No sex/infrequent sex	7.1	(5.3, 8.8)
Lack of access/too far	5.1	(3.4, 6.6)
Menopausal/infecund	4.8	(3.1, 6.4)
Inconvenient to use	4.4	(3.0, 5.7)
Religious prohibition	2.5	(1.4, 3.5)
Other	2.3	(1.2, 3.4)
Not married	2.0	(0.9, 3.0)
Others opposed	1.2	(0.3, 2.0)
Respondent opposed0	1.1	(0.4, 1.7)
Don’t know	0.1	(<0.1, 0.3)

N=1140, missing=260.

### Proximate determinants of fertility

**Figure 5:**
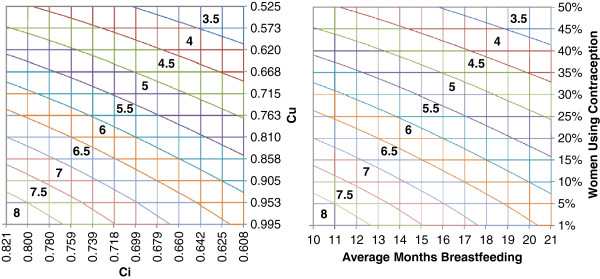
**Expected fertility rate under scenarios of contraceptive prevalence and breastfeeding.** Both figures show the relationship between contraceptive prevalence, average length of breastfeeding, and fertility. The left figure show these relationships in terms of fertility indices; the right figure shows these relationships in terms of prevalence. Values in the color bands correspond with different levels of TFR. Currently, Idjwi is positioned in the bottom left corner with 8.3 TFR, an estimated 1% contraceptive prevalence (*Cu*=0.991), and average length of total breastfeeding 10.4 months (*Ci*=0.812). If an interim goal is to reduce TFR to 6 births per woman, this figure outlines a range of scenarios to achieve that goal including increasing contraceptive prevalence to 30% (*Cu*=0.715), extending average length of breastfeeding to 21 months (*Ci*=0.608), or some combination of both such as extending average length of breastfeeding to 15 months (*Ci*=0.718) and providing contraception to 20% (*Cu*=0.810) of the Island’s sexually active fecund women.

Using the proximate determinants framework, we modeled Idjwi’s TFR to be 9.86, which is greater than TFR measured by our survey (8.3). This is likely due to under-estimation of TFR in our sample, as well as some imprecision in our estimates of proximate determinants. Despite these challenges, the model adequately captures the *relative* impact of each proximate determinant on fertility, which we report (Figure 5). Of the five proximate determinants, fertility is most influenced by the proportion of sexually active women who are unable to become pregnant (*Cx*=0.668), length of breastfeeding (*Ci*=0.812), and the proportion of women who are sexually active (*Cf*=0.874). Induced abortion (*Ca*=1) and contraceptive prevalence (*Cu*=0.991) currently play little role in determining fertility. These figures coincide with settings where there are no limits on fertility and contraceptive use is virtually zero [38].

Figure 5 presents different scenarios for reducing TFR on Idjwi by extending average length of breastfeeding and contraceptive prevalence. For example, the desired TFR of 6 could be achieved by extending average length of breastfeeding from 10 to 21 months, increasing contraceptive prevalence to 30%, or some combination of both (e.g., extending average length of breastfeeding to 15 months and providing contraception to 20% of the Island’s sexually active fecund women). Figure 4 suggests that a modest increase in contraceptive prevalence to 15% could reduce unmet need for family planning by 8% (from 54.8% to 46.6%).

## Discussion

Our results derive from what we understand to be the first representative household survey of health on Idjwi Island, DRC. We found very high fertility and extremely limited access to modern contraceptives. More than half the women on Idjwi had an unmet need for contraception, and over two thirds had an expressed desire to use contraceptives. The vast majority of women were uninformed about the advantages of having smaller families, unfamiliar with options to control fertility, and socially disempowered to make decisions about their own reproductive health. Using a proximate determinates model of fertility, we predicted that modest increases in contraceptive prevalence and extended periods of breastfeeding could meet women’s demand for family planning by reducing the TFR to 6 or fewer children per woman, while also curbing population growth and preventing further pressure on the environment.

From a public health perspective, there is a strong imperative to translate findings to practical solutions [39]. Here, we discuss several ways to meet women’s individual needs for family planning to achieve personal fertility preferences, and reduce the fertility rate at a population level. We use perspectives from key informant interviews and focus groups on Idjwi, as well as the scientific literature, to formulate specific recommendations in the areas of women’s empowerment and improved health services.

### Perspectives from Idjwi

In interviews, local leaders across sectors express that high fertility is a consequence of poverty and gender roles. Most households wish to send their sons and daughters to school, but cannot afford to send everyone. Households often choose to send sons to school because sons traditionally stay with the family to protect and provide for relatives, while daughters are married off to other families. As a result, girls receive less education than boys, have fewer career prospects, and marry early into a life of disempowerment and high fertility. To local leaders, empowering women and reducing fertility requires lifting households out of poverty so that all children can attend school. The critical ways to reduce household poverty on Idjwi, according to local leaders, is to afford residents greater access to credit, create cash crop cooperatives that sell to global markets, and provide education and supplies to help farmers increase their crop yield. Ultimately, this is a long-term path toward sustained income growth, higher education enrollment, women’s empowerment, and decreased fertility.

Local officials agree on several short-term solutions for empowering women and decreasing unmet need for family planning. First, many informants advocated for skills-training programs for uneducated, married women (e.g., nursing, cooking, tailoring, and typing). Programs would provide women with a source of income, granting them greater control in their relationships and health care decisions. Second, in the absence of Congolese support, local leaders advocate for aid organizations to provide universal free access to contraceptives and education around family planning. To decrease unmet need on Idjwi, they argue that women and men need the opportunity to learn about the use and safety of contraceptives, and have the freedom to choose a contraceptive method regardless of income.

### Perspectives from the scientific literature

Evidence from countries that have transitioned from high to low fertility suggest that reductions in child mortality are often a prerequisite for lowering fertility preferences [21], although this is not a necessary condition (e.g., France observed sustained reductions in fertility prior to mortality declines) [40]*.* We do not report child mortality statistics here because we have evidence of under-reporting of child deaths in our survey. Assuming that under-five mortality on Idjwi is at least as high as the rest of South Kivu province at 186 deaths per 1000 live births [12], the literature suggests that improving child survival is key to reducing the TFR.

Education, particularly of young women, empowers them to meet their desired fertility levels [41,42], and in the long term, will likely influence them to desire smaller families [41]. Women who can control their own fertility are positioned to make decisions about their own education, work, and health care, and can develop social roles outside of raising children. Education for women also has a positive impact on their children’s health. Women who have at least a secondary education generally wait longer to have their first pregnancy and space their births, resulting in healthier children [4]. Education of women is also associated with lower rates of domestic violence and greater female empowerment [43].

### Recommendations

Based on our results and perspectives from the literature and the field, we recommend interventions that empower women with education; increase geographic, economic, and social access to family planning for women and men; promote extended periods of breastfeeding; and reduce child mortality. We call upon implementers of family planning programs and policies to be cognisant of Idjwi’s social and economic inequalities, and to attempt equity and fairness in the provision of family planning services by reaching out specifically to poor women and Batwa women. We also suggest that men are engaged with reproductive health information and services.

#### Women's empowerment

A suite of programs are needed to empower women to obtain education and economic opportunities, and to minimize social inequalities and violence against women. We recommend (1) providing legal support to women to seek justice against perpetrators of sexual violence; (2) promoting discussions and practice of gender equality in community institutions such as churches and schools; (3) creating safe spaces for men to investigate masculinity and to practice respectful behaviors toward women; and (4) integrating gender justice programs into classrooms and afterschool activities [44].

Our results indicate that extending the average period of breastfeeding by just a few months can reduce total fertility on Idjwi. Breastfeeding education campaigns empower women who wish to control their birth spacing but who are unable to access, or prefer not to use, modern contraception, with the important additional benefit of improving child nutrition and survival [26]. New and expecting mothers can be reached during antenatal care visits, via public campaigns, and at facility-based deliveries with information about the effects of extended breastfeeding on birth spacing and child health [45].

#### Health services

Family planning services require access to contraceptives and trained medical personnel. In interviews, local leaders affirmed they would welcome partner organizations to improve quality and quantity of health services. We recommend that organizations already working in eastern DRC approach Idjwi clinic administrators about providing contraceptives and training personnel.

In addition to making family planning services available, we recommend taking advantage of all interactions with female patients to inform them about contraception and breastfeeding. Our survey indicated that over 90% of women visited a health center or hospital on Idjwi during the last year (not shown)—and yet, large portions of women with an unmet need for contraception cited lack of knowledge about how contraceptives work, contraceptive options, and where to obtain contraceptives as reasons for not using contraception (Table 4).

Finally, we recommend the development of a community health worker program that offers basic family planning, and maternal and child health services based out of Idjwi’s health centers, ensuring that community health workers are compensated and supported with adequate resources to promote worker quality and retention [46]. In other low-resource settings, paid community health workers play an integral role in educating the public about family planning, delivering contraceptives, and providing basic child health services such as life-saving oral rehydration therapy to children with diarrhea [47,48]. Community health programs that involve men through group discussions and provision of health services show greater improvements in women’s reproductive health outcomes than programs that provide services to women alone [23].

### Strengths

Our sampling approach resulted in a representative sample of Idjwi. By following gold-standard practices for training local interviewers and conducting respectful, standardized interviews, we built trust in the communities we sampled and collected a rich dataset to inform Idjwi health policies and programming. We also conducted extensive ethnographic data collection to provide local context for the results, and included community perspectives in our recommendations.

### Limitations

The internal review board required that only “adults” be interviewed, and the legal age of adulthood in the DRC is 18. This restriction may create biases, since women on Idjwi marry, start households, and begin having children before the age of 18, and teen mothers in similar settings have greater reproductive health needs and poorer social, economic, and health outcomes than adults [49]. Although the statistics presented here are bleak, reproductive health needs on Idjwi may be even greater once the outcomes of young women are included. Yet, our findings based on adults aged 18 and above, and the recommendations put forth, are likely to be equally valid for young adults. Additionally, this study did not interview men about their fertility preferences, or their sexual health attitudes, knowledge, and behaviour which is important for designing interventions that include men.

## Conclusions

Our findings present a fresh look at critical population problems faced on Idjwi. With extremely high fertility and virtually no availability of contraceptives, there is much room for improvement in family planning services. By exposing Idjwi’s needs and challenges on the basis of solid evidence, we expect that more attention will be devoted to the empowerment of women to control their own fertility, and that organizations working in eastern DRC will extend their support to programs that improve Idjwi’s healthcare system, including family planning services, child survival, and women's empowerment. This study provides a framework for quantifying demand for family planning and using a proximate determinates model of fertility to evaluate the potential effects of various family planning intervention scenarios in a low-resourced environment.

## Competing interests

The authors declare that they have no competing interests.

## Authors' contributions

DRT and MBH conducted the statistical analysis and drafted the manuscript. MCC provided guidance in the statistical analysis and edited the manuscript. PGG provided early guidance on the study design and edited the manuscript. All authors read and approved the final manuscript.

## Pre-publication history

The pre-publication history for this paper can be accessed here:

http://www.biomedcentral.com/1471-2458/12/959/prepub
